# Valvular Heart Disease Mortality in the United States, 2023

**DOI:** 10.1016/j.jacadv.2026.102749

**Published:** 2026-04-20

**Authors:** Rebecca C. Woodruff, Xin Tong, Essi M. Havor, Omoye Imoisili, Nilay S. Shah, Adam S. Vaughan, Fleetwood Loustalot, Fátima Coronado, Susan A. Carlson

**Affiliations:** a Division for Heart Disease and Stroke Prevention, National Center for Chronic Disease Prevention and Health Promotion, CDC, Atlanta, Georgia, USA; b United States Public Health Service Commissioned Corps, Rockville, Maryland, USA; c Epidemic Intelligence Service, Centers for Disease Control and Prevention (CDC), Atlanta, Georgia, USA; d Division of Cardiology, Department of Medicine, Northwestern University Feinberg School of Medicine, Chicago, Illinois, USA

**Keywords:** adults, epidemiology, heart valve disease, mortality, United States, vital statistics

Innovation in clinical and public health approaches to address valvular heart disease could improve population health in the United States. Valvular heart disease refers to damage to any of the heart’s 4 valves that regulate blood flow through the heart’s chambers. In 2022, the U.S. Congress passed the Cardiovascular Advances in Research and Opportunities Legacy (CAROL) Act, which directs funding for research and awareness campaigns, to improve diagnostic technologies and surgical options for valve repair or replacement.^1^ Comprehensive mortality surveillance for valvular heart disease could help guide these investments. Although prior reports have described trends in mortality rates from valvular heart disease resulting from a degenerative process that is listed as the underlying cause of death,^2^^,^^3^ limited information is available on the total number of deaths involving valvular heart disease, including from rheumatic or congenital etiologies or from valvular heart disease listed as a contributing cause of death. The analysis describes death counts and proportions in the U.S. in 2023 related to valvular heart disease categories using a comprehensive definition among the overall population and by age group.

**What is the clinical question being addressed?**

How many deaths related to degenerative, rheumatic, or congenital valvular heart disease occurred in the United States in 2023?

**What is the main finding?**

Nearly 67,000 deaths related to valvular heart disease occurred, representing approximately 2% of overall deaths.

## Methods

Death counts in 2023 among residents of 50 U.S. states and the District of Columbia were obtained from the Multiple Cause of Death Files from the National Center for Health Statistics’ National Vital Statistics System. Deaths with valvular heart disease listed as the single underlying cause of death or among the 20 possible contributing causes of death were identified using International Classification of Disease-10th Revision (ICD-10) codes adapted from prior work:^3^ rheumatic valvular heart disease (I05–I09); degenerative valvular heart disease (I34–I38); or congenital valvular heart disease (Q22–Q23). Valvular heart disease deaths were further classified by the specified valve and category (Table 1). Death counts and proportions were presented separately by cause of death type (ie, valvular heart disease as the underlying vs contributing cause of death) and by age group (ie, adults aged ≥18 years and infants and children aged <18 years). Death counts and proportions are not necessarily mutually exclusive as ICD-10 codes can specify multiple affected valves or disease processes and multiple ICD-10 codes can be listed as contributing causes of death. Measures of uncertainty and inferential statistics were not used, as these data reflect national death counts. Data were analyzed in SAS (version 9.4, SAS Institute). Institutional Review Board review was not required for this analysis of publicly available data.

**Table 1:** Characteristics of Valvular Heart Disease Deaths—United States, National Vital Statistics System, 2023

	Death Counts Among All Ages			Overall Death Counts	
	By Cause of Death Type			By Age Group	
	Overall^a^	Underlying^a^	Contributing^a^	<18 Years^b^	≥18 Years
Overall	66,984 (100)	28,504 (100)	38,480 (100)	565 (100)	66,419 (100)
Etiology^c^					
Degenerative	57,802 (86.3)	23,607 (82.8)	34,195 (88.9)	63 (11.2)	57,739 (86.9)
Rheumatic	9,301 (13.9)	4,283 (15.0)	5,018 (13.0)	35 (6.2)	9,266 (14.0)
Congenital	1,091 (1.6)	614 (2.2)	477 (1.2)	490 (86.7)	601 (0.9)
Specified valve^d^					
Aortic	40,102 (59.9)	16,956 (59.5)	23,146 (60.2)	107 (18.9)	39,995 (60.2)
Mitral	13,568 (20.3)	5,987 (21.0)	7,581 (19.7)	73 (12.9)	13,495 (20.3)
Tricuspid	3,202 (4.8)	1,178 (4.1)	2,024 (5.3)	48 (8.5)	3,154 (4.7)
Pulmonary	116 (0.2)	27 (0.1)	89 (0.2)	29 (5.1)	87 (0.1)
Other or unspecified	14,579 (21.8)	5,815 (20.4)	8,764 (22.8)	398 (70.4)	14,181 (21.4)
Valvular heart disease categories^e^					
Aortic stenosis	30,541 (45.6)	13,115 (46.0)	17,426 (45.3)	52 (9.2)	30,489 (45.9)
Endocarditis, valve unspecified	13,099 (19.6)	5,178 (18.2)	7,921 (20.6)	16 (2.8)	13,083 (19.7)
Unspecified aortic valve disease	7,468 (11.1)	2,967 (10.4)	4,501 (11.7)	20 (3.5)	7,448 (11.2)
Mitral insufficiency	6,970 (10.4)	2,707 (9.5)	4,263 (11.1)	15 (2.7)	6,955 (10.5)
Unspecified mitral valve disease	5,138 (7.7)	2,407 (8.4)	2,731 (7.1)	22 (3.9)	5,116 (7.7)
Aortic insufficiency	2,657 (4.0)	984 (3.5)	1,673 (4.3)	41 (7.3)	2,616 (3.9)
Tricuspid insufficiency	2,197 (3.3)	700 (2.5)	1,497 (3.9)	9 (1.6)	2,188 (3.3)
Mitral stenosis	1,813 (2.7)	911 (3.2)	902 (2.3)	39 (6.9)	1,774 (2.7)
Unspecified tricuspid valve disease	954 (1.4)	447 (1.6)	507 (1.3)	18 (3.2)	936 (1.4)
Hypoplastic left heart syndrome	363 (0.5)	269 (0.9)	94 (0.2)	319 (56.5)	44 (0.1)
Tricuspid stenosis	69 (0.1)	32 (0.1)	37 (0.1)	23 (4.1)	46 (0.1)
Unspecified pulmonary valve disease	48 (0.1)	14 (0.05)	34 (0.1)	16 (2.8)	32 (0.05)
Pulmonary insufficiency	35 (0.1)	6 (0.02)	29 (0.1)	0 (0)	35 (0.1)
Pulmonary stenosis	33 (0.05)	7 (0.02)	26 (0.1)	13 (2.3)	20 (0.03)

Counts and percentages for etiologies, specified valves, and valvular heart disease categories do not always sum to total because multiple causes of death can be listed on death certificates.

a Overall death counts represent deaths that list valvular heart disease on the death certificate as the single underlying cause of death or among the 20 possible contributing causes of death. Because death counts for overall deaths and contributing causes of death are not mutually exclusive, death counts and proportions may not sum to total.

b Pediatric deaths include all deaths among infants and children aged <18 years. including infant deaths during the neonatal (<28 days) and postneonatal (28-364 days) periods. Most pediatric deaths were among infants aged <1 year (408, 72.2%).

c Deaths were categorized by ICD-10 codes for degenerative heart valve disease (I34-I38), rheumatic heart disease (I05-I09), and congenital heart valve disease (Q22-Q23).

d Deaths were classified by specified valve based on the following ICD-10 codes: aortic valve (I06, I08.0, I08.2, I08.3, I35, Q23.0, I23.1, I23.8-Q23.9), mitral valve (I05, I08.0, I08.1, I08.3, I34, Q23.2, Q23.3, Q23.8, Q23.9), tricuspid valve (I07, I08.1, I08.2, I08.3, O36, Q22.4, Q22.8, Q22.9), pulmonary valve (I37, Q22.0, Q22.1, Q22.2, Q22.3), and other or unspecified valvular heart disease (I08.8, I08.9, I09, I38, Q22.5, Q22.6, Q23.4).

e Categories were defined by ICD-10 codes as follows: aortic stenosis (I06.0, I06.2, I35.0, I35.2, Q23.0), endocarditis (I38), unspecified aortic valve disease (I06.8, I06.9, I08.0, I08.2, I08.3, I35.8, I35.9, Q23.8, Q23.9), mitral insufficiency (I05.1, I05.2, I34.0, I34.1, unspecified mitral valve disease (I05.8, I05.9, I08.0, I08.1, I08.3, I34.8, I34.9, Q23.8, Q23.9), aortic insufficiency (I06.1, I06.2, I35.1, I35.2, Q23.1), tricuspid insufficiency (I07.1, I07.2, I36.1, I36.2), mitral stenosis (I05.0, I05.2, I34.2, Q23.2, Q23.3), unspecified tricuspid valve disease (I07.8, I07.9, I08.1, I08.2, I08.3, I36.8, I36.9, Q22.8, Q22.9), hypoplastic left heart syndrome (Q23.4), tricuspid stenosis (I07.0, I07.2, I36.0, I36.2, Q22.4), unspecified pulmonary valve disease (I37.8, I37.9, Q22.0, Q22.3), pulmonary insufficiency (I37.1, I37.2, Q22.2), and pulmonary stenosis (I37.0, I37.2, Q22.1).

## Results

In 2023, 3,091,127 all-cause deaths occurred in the United States; 66,984 (2.2%) were related to valvular heart disease (Table 1). Most deaths related to valvular heart disease were among adults aged ≥18 years (66,419, 99.2%); of 565 deaths among those aged <18 years, 408 (72.2%) were among infants aged <1 year. Most valvular heart disease deaths were among females (34,201, 51.1%) and non-Hispanic White decedents (55,745, 83.2%).

Among valvular heart disease deaths, 28,504 (42.6%) listed valvular heart disease as the underlying cause of death and 38,480 (57.4%) listed valvular heart disease as a contributing cause of death. Most (57,802, 86.3%) valvular heart disease deaths involved degenerative disease; 9,301 (13.9%) involved rheumatic disease and 1,091 (1.6%) involved congenital disease. The aortic valve was the most frequently specified (40,102, 59.9%), followed by the mitral valve (13,568, 20.3%), tricuspid valve (3,202, 4.8%), and pulmonary valve (116, 0.2%). In 2,722 (4.1%) deaths, multiple valves were specified, and in 4,579 (21.8%) deaths, ICD-10 codes did not specify a valve. The most frequently documented valvular heart disease categories were aortic stenosis (30,541, 45.6%), endocarditis with unspecified valve (13,099, 19.6%), unspecified aortic valve disease (7,468, 11.1%), mitral insufficiency (6,970, 10.4%), unspecified mitral valve disease (5,138, 7.7%), aortic insufficiency (2,657, 4.0%), tricuspid insufficiency (2,197, 3.3%), mitral stenosis (1,813, 2.7%), and unspecified tricuspid valve disease (954, 1.4%); all other subtypes were specified in <1% of overall valvular heart disease deaths.

Valvular heart disease deaths among adults primarily involved degenerative (57,739, 86.9%) or rheumatic (9,266, 14.0%) disease. The top 5 most frequently occurring categories among adults were aortic stenosis, endocarditis, unspecified aortic valve disease, mitral insufficiency, and unspecified mitral valve disease. Valvular heart disease deaths among infants and children primarily involved congenital disease (409, 6.7%). The top 5 most frequently occurring categories among infants and children were hypoplastic left heart syndrome, aortic stenosis, aortic insufficiency, mitral stenosis, and tricuspid stenosis.

## Discussion

Nearly 67,000 deaths in the United States were related to valvular heart disease in 2023, accounting for approximately 2% of all-cause mortality. Most deaths occurred among adults and were primarily from degenerative etiology, whereas deaths among children were predominantly due to congenital causes. By using an expanded surveillance definition that includes valvular heart disease of rheumatic or congenital etiologies and considers contributing causes of death, we detected more than double the number of valvular heart disease deaths than a definition using underlying cause of death alone.^2^^,^^3^ Most of these additional deaths involved valvular heart disease listed as a contributing cause of death, which might reflect acute, chronic, or surgically treated conditions that were determined to be clinically relevant to the death, even if they were not part of the causal chain of events primarily responsible for the death.

Improvements in surveillance of valvular heart disease-related mortality could strengthen population health monitoring and guide clinical and public health strategies. Importantly, 5 of the 10 most frequently recorded valvular heart disease categories were coded as deaths from unspecified valvular disease. Nonspecific coding limits accurate assessment of mortality by valve type or disease processes, potentially leading to underestimation of deaths involving disease categories with established, guideline-directed therapies, such as transcatheter aortic valve replacement for aortic stenosis.^2^ Incomplete characterization can also obscure clinical quality improvement opportunities, research priorities, and research allocation by masking mortality related to valve-specific disease processes. Under-reporting or misclassification of valvular heart disease on death certificates, particularly if differential across diagnostic categories or population groups, is a known limitation of vital records data and of this analysis.^4^ Improving the specificity of cause of death reporting, potentially through enhanced training for death certificate certifiers, could improve the accuracy and utility of surveillance for valvular heart disease mortality and other causes of death.

Robust surveillance of valvular heart disease mortality could inform clinical and public health efforts. Future efforts that monitor age-adjusted mortality rates for valvular heart disease over time and across demographic and geographic groups can facilitate trend analyses and identify populations at greatest risk for valvular heart disease mortality while accounting for differing population sizes and age structures. Comprehensive surveillance of valvular heart disease-related mortality could help align funding to expand tailored awareness-raising campaigns and efforts to promote early detection and expanded access to specialized care with populations experiencing the greatest burden of valvular heart disease. Through the CAROL Act, the Centers for Disease Control and Prevention has developed toolkits for health care professionals, partners, and the public to promote educational materials to encourage early identification of valvular heart disease^5^ and the National Heart, Lung, and Blood Institute is funding research and improve the understanding, diagnosis, and treatment of valvular heart disease. The CAROL Act offers an important framework to translate surveillance findings into actionable strategies, including earlier detection, more precise diagnosis, and expanded access to effective therapies.^1^

## Funding support and author disclosures

The findings and conclusions of this report are those of the authors and do not necessarily represent the official position of the Centers for Disease Control and Prevention. The authors have reported that they have no relationships relevant to the contents of this paper to disclose.
